# Prevalence and Determinants of Depressive Symptoms in Older Adults Across Europe: Evidence from SHARE Wave 9

**DOI:** 10.3390/jcm14155340

**Published:** 2025-07-29

**Authors:** Daniela Melo, Luís Midão, Inês Mimoso, Leovaldo Alcântara, Teodora Figueiredo, Joana Carrilho, Elísio Costa

**Affiliations:** 1 RISE-Health, Competence Centre for Active and Healthy Ageing, Faculty of Pharmacy, University of Porto, Rua Jorge de Viterbo Ferreira 228, 4050-313 Porto, Portugal; dfmelo@ff.up.pt (D.M.); lmidao@ff.up.pt (L.M.); ifmimoso@ff.up.pt (I.M.); leovaldoalcantara@gmail.com (L.A.); tgfigueiredo@ff.up.pt (T.F.); jcarrilho@ff.up.pt (J.C.); 2 School of Medicine and Biomedical Sciences, University of Porto, Rua Jorge de Viterbo Ferreira 228, 4050-313 Porto, Portugal

**Keywords:** late-life depression, ageing, prevalence, SHARE, mental health

## Abstract

**Background/Objectives**: The rapid ageing of the European population presents growing challenges for mental health, highlighting the need to identify factors that can prevent or delay psychological decline and promote a higher quality of life in later life. This study aims to provide an updated and comprehensive overview of mental health among older adults in Europe by examining the prevalence of depressive symptoms and identifying key associated factors. **Methods**: We analysed data from individuals (*n* = 45,601) aged 65 years and older across 27 European countries and Israel who participated in Wave 9 of the Survey of Health, Ageing and Retirement in Europe (SHARE). This study assessed the prevalence of depressive symptoms, which were evaluated using the EURO-D scale (score range: 0–12), with a cut-off of ≥4 indicating clinically relevant symptoms. It also explored associations with sociodemographic characteristics, physical health, behavioural factors, social participation, internet skills and living conditions. **Results**: Our findings confirm that depressive symptoms remain highly prevalent among older adults in Europe, with 35.1% of women and 21.5% of men affected, reflecting persistent gender disparities in mental health. Depression in later life was significantly associated with poor physical health, loneliness and lower quality of life. Conversely, moderate involvement in grandchild care and in social participation emerged as potential protective factors. **Conclusions**: Late-life depression has substantial implications for both mental and physical well-being. Our findings suggest that social integration, gender related factors and physical health are closely associated with depressive symptoms in older adults. These associations highlight the importance of considering these domains when designing interventions and policies aimed at promoting mental health in ageing populations.

## 1. Introduction

A significant accomplishment of the last century has been the increase in average life expectancy due to advancements in healthcare, technology and overall living conditions [1,2]. However, the gap between life expectancy and healthy life expectancy continues to grow in Europe, with many individuals living considerable years in poor health [3]. The increase in life expectancy and the declining fertility rates has led to the rapid ageing of the European population, which presents significant challenges for public health, such as a growing prevalence of mental health issues among older adults [4,5].

According to the World Health Organization [6], mental health is more than just the absence of mental disorders, it encompasses both the prevention of negative mental states and the promotion of positive psychological well-being. Depression remains a major mental health concern in older adults. It is estimated that over one-third of the older population experiences depression [7], but this condition is often underrecognized and undertreated in older individuals [8,9,10,11]. When left untreated or inadequately managed, late-life depression is associated with higher mortality rates, dementia and suicide [10,12].

Social isolation and loneliness comprise one of the main factors contributing to the risk of depressive symptoms in aged individuals, especially in high-income countries [13,14,15]. Ageing often leads to reduced social participation due to declining physical and psychological functioning [16,17]. Compared to younger adults, seniors usually have smaller social networks composed mainly of close family members or long-term friends, and the increasing tendency to live alone or separate from family increases the risk of loneliness [18,19,20].

Societal changes, including evolving family structures and intergenerational dynamics, limit meaningful social interactions among older adults, thereby increasing loneliness [8,18,21,22]. Participation in intergenerational activities, such as those involving grandchildren, has been associated with enhanced emotional well-being and reduced depressive symptoms [23,24,25].

In this context, digital technologies have emerged as a potential means to reduce social isolation and promote mental health among seniors [26,27]. Research indicates [28,29,30] that internet use can have positive effects on mental health by reducing loneliness and depressive symptoms in seniors. However, barriers such as limited digital literacy, restricted access to technology [31,32,33], mobility limitations [34], hearing and vision impairment [35], rural residency [36] and socioeconomic disparities [8,37] still hinder the effective use of such tools.

Thus, understanding these diverse and multifaceted events is essential to identify factors that prevent or delay mental health decline and that promote a higher quality of life for older adults, maintaining the long-term sustainability of European healthcare and financial systems [5].

Previous research often focuses on isolated variables when evaluating mental health in the older population. Thus, this study analyses multiple interconnected factors including sociodemographic characteristics, physical health, behavioural factors, social participation, internet skills and living conditions. Our aim is to provide an updated and comprehensive overview of the mental health landscape in Europe, using data from Wave 9 of the Survey of Health, Ageing and Retirement in Europe (SHARE), by examining the prevalence of depressive symptoms among older adults across 27 European countries and Israel and identifying the main associated factors.

## 2. Materials and Methods

**Figure 1:**
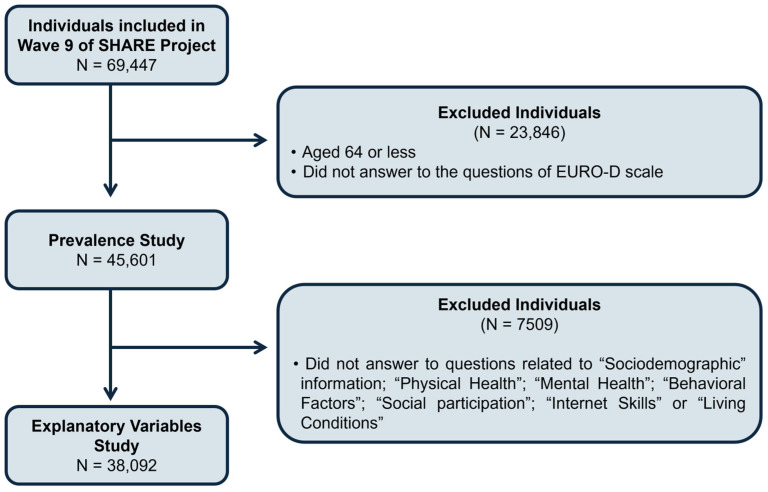
Flow diagram illustrating the selection process of participants for the prevalence and explanatory variables analysis.

This work used data from the SHARE Wave 9 database, which is an international and multidisciplinary database of detailed information about demographics, health, socioeconomic status and social and family networks from representative samples of community-based populations from 27 European countries (Austria, Belgium, Bulgaria, Croatia, Cyprus, the Czech Republic, Denmark, Estonia, Finland, France, Germany, Greece, Hungary, Italy, Latvia, Lithuania, Luxembourg, Malta, the Netherlands, Poland, Portugal, Romania, Slovakia, Slovenia, Spain, Sweden and Switzerland) and Israel. For statistical analysis proposes, we grouped these countries into four regions: northern (Denmark, Estonia, Finland, Latvia, Lithuania and Sweden), southern (Croatia, Cyprus, Greece, Italy, Malta, Portugal, Slovenia, Spain and Israel), western (Austria, Belgium, France, Germany, Luxembourg, Netherlands and Switzerland) and eastern (Bulgaria, Czech Republic, Hungary, Poland, Romania and Slovakia). This project is an important source of data for ageing research in Europe [38]. SHARE data was collected through computer-assisted personal interviewing conducted face-to-face by trained interviewers, and participants were recruited from the general population based on specific eligibility criteria: individuals aged 50 years and older who resided regularly in the participating SHARE country. Exclusions applied to individuals who were incarcerated, hospitalized for the entire fieldwork period, had moved abroad or to an unknown address or who could not speak the national language(s). Wave 9 data were collected between October 2021 and September 2022 from 69,447 individuals, aged between 20 and 106 years. For this work, we analysed individuals aged 65 years or older, who were categorised into three groups: 65–74 years, 75–84 years and 85 years or older (Figure 1).

### 2.1. Prevalence of Depressive Symptoms

Depressive symptoms were evaluated using the EURO-D scale that measures current levels of depression based on 12 items: depressed mood, pessimism, suicidality, guilt, sleep, interest, irritability, appetite, fatigue, concentration, enjoyment and tearfulness. Every item is scored 1 if present and 0 if absent. The final scores ranged from 0 (“not depressed”) to 12 (“highly depressed”) and were dichotomised into “Without depressive symptoms” (scores < 4) and “With depressive symptoms” (scores ≥ 4) [39,40]. The specific questions of the EURO-D scale can be found in the Supplementary Materials section.

### 2.2. Explanatory Variables

As a result of the large amount of information available in the SHARE database, we were able to evaluate a diverse set of putative explanatory variables in this study. These variables were organised into seven groups: Sociodemographic, Physical Health, Mental Health, Behavioural Factors, Social Participation, Internet Skills and Living Conditions. Detailed information about questions and answer options for each variable can be obtained from the Supplementary Materials section.

#### 2.2.1. Sociodemographic

The variables “Gender”, “Age”, “Marital Status”, “Years of Education”, “Shortage of Money” and “Job Situation” were included in this group. “Gender” was identified as either male or female. “Age” was calculated by subtracting the participants’ reported birth year from 2022 and grouped into three categories, 65–74 years, 75–84 years and 85 years or older, as previously defined [40]. “Marital Status” and “Job Situation” were evaluated based on self-reported information and categorised accordingly, as described in [8,40]. The education level (“Years of Education”) was measured by the total number of years spent in full-time education and categorised into four levels [40]. Socioeconomic status (“Shortage of Money”) was assessed through a question about financial difficulties impacting daily activities, in line with previous research [40].

#### 2.2.2. Physical Health

This group incorporated the following variables: “Number of Chronic Diseases”, “Number of Limitations in Activities of Daily Living (ADLs)”, “Number of Limitations in Instrumental Activities of Daily Living (iADLs)”, “Limitation in Activities because of Health”, “Hearing”, “Vision”, “Pain”, “Receive Professional Health Services” and “Hospital Stays in the Last 12 months”. The “Number of Chronic Diseases”, “Limitation in Activities because of Health”, “Hearing”, “Vision” and “Pain” variables were evaluated based on self-reported diagnoses by each individual and categorised according to previous studies [35,40]. The number of ADLs and iADLs were calculated by summing reported difficulties in different tasks and recoded into three levels, in line with previous research [40]. “Hospital Stays in the Last 12 months” and “Received Professional Health Services” were recorded as binary variables, as previously described [40].

#### 2.2.3. Mental Health

Besides the depressive symptoms, mental health in older adults was also addressed by self-evaluation questions about the quality of life, well-being and loneliness. “Quality of Life and Well-Being” was determined through the Control, Autonomy, Self-realisation and Pleasure (CASP-12) scale, a 12-item self-assessment questionnaire that measures quality of life [41,42]. Each question is rated from 1 (never) to 4 (often), and the total score ranges from 12 to 48, with higher scores meaning better quality of life. “Loneliness” was evaluated by the Three-Item Loneliness Scale, which measures indirect loneliness based on questions about companionship, feeling left out and isolation [43,44]. Total scores range from 3 to 9, and the final answers were grouped into two categories [8]: “Not lonely” for scores between 3 and 5, and “Lonely” for scores of 6 or higher.

#### 2.2.4. Behavioural Factors

The variables “Physical Inactivity”, “Alcohol Intake” and “Ever Smoked Daily” were included in this group. “Physical Inactivity” was assessed through questions about engaging in vigorous or moderate physical activity, and it was recoded into two categories, “Engage in vigorous or moderate physical activity” and “Never engage in vigorous or moderate physical activity”, as previous defined [8]. “Ever Smoked Daily” and “Alcohol Intake” were recorded as binary variables [40].

#### 2.2.5. Social Participation

To identify older adults’ social participation in activities, social networks and intergenerational relationships, the variables “Number of Social Activities”, “Satisfaction with Social Activities”, “Network Satisfaction” and “Looking after Grandchildren” were assessed and categorised as described in [21,22]. The “Number of Social Activities” was calculated by asking the participants which social activities they had engaged in the last year, and answers were grouped into three levels. “Satisfaction with Social Activities” and “Network Satisfaction” were evaluated on a scale from 0 to 10, where 0 means completely dissatisfied and 10 means completely satisfied. “Looking after Grandchildren” was assessed based on questions about having grandchildren and looking after them regularly or occasionally, and answers were grouped into three categories.

#### 2.2.6. Internet Skills

Internet skills were determined by asking the participants if they used the internet for several purposes during the last week. Responses were recorded as “Yes” and “No”, as previously described [33].

#### 2.2.7. Living Conditions

Living conditions were evaluated using two variables: “Area of Building” and “Type of Building”. Both variables were based on self-reported information from each participant about the place where they live and categorised according to previous research [40].

### 2.3. Statistical Analysis

IBM SPSS Statistics 29 for Windows (SPSS Inc., Chicago, IL, USA) software was used for statistical analysis. A descriptive analysis was conducted to estimate the proportion of individuals experiencing depressive symptoms across 28 countries. The age and gender standardised prevalence of depressive symptoms in each country was calculated, along with the corresponding 95% confidence intervals (95% CI). The 2013 European standard population was used to standardise all prevalence results. Considering the multilevel nature of the data, with individuals grouped within countries, a multilevel logistic regression model was employed, treating depressive symptoms as the dependent variable. Initially, multilevel univariable logistic regression models were conducted to evaluate the relationship between each individual covariate and depressive symptoms (unadjusted model). Given the multilevel structure and the focus on the final multivariable multilevel model where covariates were mutually adjusted, we did not apply formal correction for multiple comparisons in the univariable analyses. Covariates found to be statistically significant were subsequently entered into a multivariable multilevel logistic regression model (adjusted model) [45,46]. To assess whether the association between explanatory variables and depressive symptoms varied by age group or by cultural differences between European regions, stratified analysis (bivariate logistic regression) was performed to evaluate the association between them across different age groups (Table S1) and different regions (Table S2). All the results were presented as odds ratios (OR) with a corresponding 95% CI. A *p* < 0.05 value was considered statistically significant.

## 3. Results

### 3.1. Prevalence Study

For this study, from all the participants in Wave 9 of the SHARE survey, we selected those aged 65 years or older who answered questions related to “Age”, “Gender” and to the EURO-D scale, which resulted in a total of 45,601 individuals (Figure 1).

**Figure 2:**
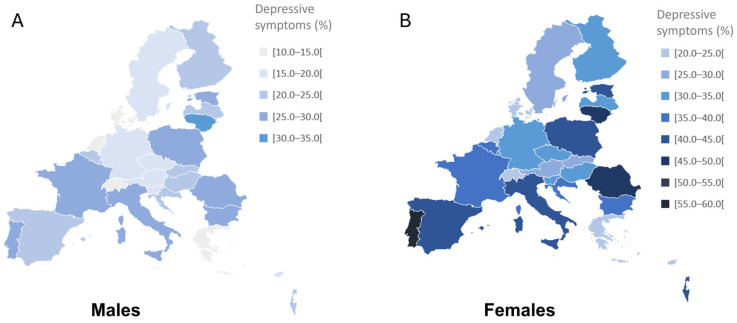
Prevalence of depressive symptoms among European older adults (≥65 years) in 27 European countries and Israel, by gender: males (**A**) and females (**B**). Data are presented as percentages.

Participants presented a mean age of 74.9 ± 7.0, and 56.7% were female. The geographical distribution of depressive symptoms was evaluated across different European countries and Israel, and the results are represented in Figure 2.

The overall prevalence of depressive symptoms was 29.2%, with prevalence rates varying from 18.4% to 44.5%. Portugal (44.5%) presented the highest prevalence of depressive symptoms, followed by Lithuania (42.6%) and Romania (38.6%), while Denmark (18.4%), the Netherlands (19.1%) and Switzerland (19.1%) showed the lowest rates (Table 1). The presence of depressive symptoms was higher in women than men (35.1% and 21.5%, respectively) for all age groups and countries, except for people over 85 years in Bulgaria (59.1% and 46.9% for males and females) and Luxembourg (27.8% and 25.0% for males and females) and people living in Cyprus between 65 and 74 years (16.8% and 15.0% for males and females). Overall, the likelihood of experiencing depressive symptoms increased with age (24.2%, 31.9% and 43.6% for people aged 65–74 years, 75–84 years and ≥85 years, respectively). This tendency was also observed in both men (17.3%, 23.2% and 34.7% for people aged 65–74 years, 75–84 years and ≥85 years, respectively) and women (29.6%, 38.3% and 49.4% for people aged 65–74 years, 75–84 years and ≥85 years, respectively).

### 3.2. Association of Depressive Symptoms with Explanatory Variables

Participants from the prevalence study who answered all the questions related to the explanatory variables were included in the evaluation of the relationship between those variables and depressive symptoms. This process resulted in a total of 38,092 participants (Figure 1) with an average age of 74.8 ± 6.9 years, and 21,838 (42.7%) were female.

Using unadjusted models across all participating countries, a significant relationship was found between depressive symptoms and most of the exploratory variables studied (Table 2). “Area of Building” was the only variable that showed no association with depressive symptoms (Table 2).

The adjusted model (Table 2) revealed a significant association with all variables in the “Sociodemographic” group except for “Job Situation”. Women were nearly two times more prone to report depressive symptoms compared to men (OR = 1.980, 95% CI: 1.862–2.106), while participants aged 85 years were less prone to report depressive symptomatology (OR = 0.834, 95% CI: 0.754–0.922). Using the adjusted model, married individuals or those in a registered partnership, compared to those who were single, were more prone to have depressive symptoms (OR = 1.188, 95% CI: 1.025–1.378). However, this association was found to be influenced by regional heterogeneity (Table S2) and by age, since a significant association between marital status and depressive symptoms was observed only among individuals aged 75 to 84 (Table S1). “Years of Education” was identified as a variable with statistical significance only for individuals with high education (OR = 1.134, 95% CI: 1.043–1.233), but the association between “Years of Education” and depressive symptoms was observed exclusively in individuals aged 75 to 84 years (Table S1) and was found to depend on regional heterogeneity (Table S2). “Shortage of Money” was significantly associated with depressive symptoms once depressive symptoms decreased when financial difficulties increased (Tables S1, S2 and Table 2).

In the “Physical Health” group, a significant relationship was observed between depressive symptoms and all studied variables (Table 2). The likelihood of reporting depressive symptoms increased with the “Number of Chronic Diseases” in the northern and western regions (Table 2 and Table S2). Number of limitations performing “iADLs” (OR = 1.325, 95% CI: 1.230–1.428), “Hearing” (OR = 1.233, 95% CI: 1.155–1.317) and “Stayed Overnight in Hospital” (OR = 1.344, 95% CI: 1.248–1.447) had a significant association with depressive symptoms. “Received Professional Services” presented a significant relation with depressive symptoms (OR = 1.099, 95% CI: 1.004–1.204) but only for participants aged 75 to 84 years old (Table S1) and in the southern region (Table S2). “Pain” was significantly associated with depressive symptoms, as higher levels of pain were correlated with increased depressive symptoms. Conversely, fewer “Limitations in Activities because of Health” were associated with lower levels of depressive symptoms. Number of limitations performing “ADLs” (OR = 1.206, 95% CI: 1.103–1.318) and “Vision” (OR = 1.211, 95% CI: 1.136–1.292) were also significantly associated with depressive symptoms but were dependent on the cultural differences between regions (Table S2).

Regarding the “Mental Health” group, individuals presenting signs of “Loneliness” reported twice the number of depressive symptoms (OR = 2.111, 95% CI: 1.967–2.267), and higher “Quality of Life and Well-Being” (OR = 0.864, 95% CI: 0.859–0.870) were correlated with lower levels of depressive symptoms (Table 2).

Among the “Behavioural Factors”, only the variable “Ever Smoked Daily” showed a significant negative relationship with depressive symptoms (OR = 0.890, 95% CI: 0.839–0.945) just for individuals between 65 and 74 years old (Table S1) and for the northern, southern and eastern regions.

All variables in the “Social Participation” group presented a significant association with depressive symptoms (Table 2). “Satisfaction with Social Activities” (OR = 0.933, 95% CI: 0.920–0.946) was negatively associated with depressive symptoms, whereas an increase in the “Number of Social Activities” corresponded to higher levels of depression only for the northern region (Table S2). “Network Satisfaction” (OR = 0.975, 95% CI: 0.955–0.995) was also negatively correlated with depressive symptoms but only for participants aged 65–74 and 75–84 (Table S1) and for the northern region. “Looking after Grandchildren” was identified as a variable with statistical significance only for individuals who had grandchildren and contact with them (OR = 1.143, 95% CI: 1.038–1.260) but only for individuals between 65 and 74 years old (Table S1) and for the southern and western regions (Table S2).

“Internet skills” showed no association with depressive symptoms, and the variable “Type of Building” revealed a positive relationship only for participants who lived in a building with three or more floors (OR = 1.136, 95% CI: 1.014–1.273), were aged 85 or more and lived in the southern, western or eastern regions (Table 2, Tables S1 and S2).

## 4. Discussion

Depressive symptoms among older adults represent a significant public health concern, particularly in ageing populations across Europe [7]. In this study, the overall prevalence of depressive symptoms in the older European and Israeli population was found to be 29.2%. This finding is consistent with recent evidence from a meta-analysis [47] as well as with a recent study [48] using multiple waves of the SHARE dataset, both of which reported a similar prevalence of depressive symptoms among older adults of approximately 28%.

We observed a higher prevalence of depressive symptoms in women compared to men across all age groups and in every country analysed (Figure 1 and Table 1). Although women generally outlive men, they also tend to report poorer overall health, including higher rates of psychological distress [49]. Our study confirmed the consistent and marked gender difference in depressive symptoms among older adults, findings that are well documented in previous research [48,50,51,52]. However, there is still limited understanding of the underlying causes of this disparity in later life, and whether the mechanisms behind it are the same as those observed in younger populations [53]. Nevertheless, previous analyses of specific EURO-D items [54,55] suggest that this disparity is driven largely by higher rates of tearfulness, depressed mood and sleep-related complaints among women. These disparities call for gender-sensitive mental health interventions.

Overall, the prevalence of depressive symptoms in participants over the age of 85 was higher for both men and women (Table 1), as previously reported [56]. However, after using the adjusted model, we observed a lower prevalence of depressive symptoms among individuals aged 85 and older (Table 2). Some studies [57,58] propose that individuals who reach very old age tend to exhibit greater physical and psychological resilience and increasingly prioritise emotionally meaningful experiences while developing more effective coping strategies, which may help to protect against depressive symptoms.

Interestingly, individuals who were married or in a relationship reported higher levels of depressive symptoms compared to those who were single for individuals aged 75 to 84 (Table 2). This contrasts with most previous findings, which generally suggest that being married or in a relationship has a beneficial effect on mental health [50,53]. However, many of these studies did not stratify by gender, despite growing evidence that the mental health impact of marriage differs between men and women, especially in older adults [59,60]. In fact, gender roles within marriage were often traditional, with women more likely to have sacrificed educational and professional opportunities to maintain the household. In contrast, men’s educational or occupational trajectories were generally unaffected by marriage. Moreover, marital status alone may not accurately reflect relationship quality: individuals in unsatisfying or conflictual relationships may experience higher levels of depressive symptoms [61].

A higher number of “Years of Education” was associated with lower depressive symptoms, as is well documented in the literature [62], but when the adjusted model was applied, individuals with higher education exhibited more depressive symptoms for participants between 75 and 84 years old (Table 2). Some studies [63] reveal that older adults with higher education may have greater life expectations regarding career, financial security or social status and that unmet expectations in later life could lead to increased psychological distress. Another hypothesis [64] is that more educated individuals may be more cognitively aware of their own functional or social decline, which could exacerbate depressive symptoms. Nevertheless, it is also important to note that both the association between depressive symptoms with “Marital Status” and “Years of Education” were influenced by cultural differences between countries, which could explain these unexpected results.

Financial status affects health in several ways, including reduced access to health care, poor health-related choices and limited opportunities for treatments, all of which contribute to an increased risk of disability in old age [65]. Consistent with these findings, we found that a higher “Shortage of Money” was associated with an increased prevalence of depressive symptoms (Table 2). Nevertheless, it is important to note that the “Job Situation”, often linked to financial capacity, was excluded from the adjusted model, since its effects were deemed insignificant after adjusting for all variables.

There is broad consensus that physical health influences mental health, even among older adults [64], and our findings support this association. Participants with poorer physical health consistently reported higher levels of depressive symptoms (Table 2). Specifically, depressive symptoms were more prevalent among those with a higher “Number of Chronic Diseases” belonging to the northern and western regions, greater limitations in “ADLs” (except for the western region) and “iADLs”, more frequent “Limitations in Activities because of Health”, increased levels of “Pain” and greater difficulties with “Vision” (except for the northern region) and “Hearing” (Table 2). Individuals aged 75 to 84 and individuals from the southern region who stated, “Receiving Professional Health Services” and participants having “Stayed Overnight in a Hospital” also reported higher levels of depressive symptoms. These findings are in line with prior research [66,67,68,69] showing that in later life, poor physical health is a significant predictor of depressive symptoms. It is important to mention that medication use can also have a significant influence on depressive symptoms; however, although data on polypharmacy are available in the SHARE database, they do not allow for detailed information on specific medications use on depressive symptoms.

Depression significantly impacts health and risk behaviours, as it can decrease or inhibit the motivation to engage in healthy habits and prioritise one’s well-being [21]. Although “Alcohol Intake” and “Physical Inactivity” did not significantly correlate with depressive symptoms, when the adjusted model was applied, we observed that individuals between 65 and 74 years old who do not smoke daily reported lower depressive symptoms (except those from the western region) (Table 2). On the other hand, a high quality of life and well-being can contribute to better health by promoting a sense of optimism that encourages individuals to adopt and maintain healthy behaviours [70]. In fact, in our study, participants who reported more depressive symptoms also tended to report a lower “Quality of Life and Well-Being” (Table 2), reinforcing the critical role of depressive symptoms as a key determinant of perceived quality of life in older populations [71].

Social isolation and loneliness are a significant threat to the mental health and well-being of older adults, as advancing age is often accompanied by a decrease in social networks and an increased likelihood of living alone, both of which heighten the risk of depression [17,22,72]. Consequently, maintaining meaningful social relationships and engaging in social activities are consistently associated with greater psychological resilience [21]. In line with this evidence, our findings show that older adults who report signs of loneliness experience twice as many depressive symptoms, while individuals who express higher satisfaction with their social networks (only the ones living in the northern region) and social activities report fewer symptoms (Table 2).

However, an unexpected finding in our study was that a higher “Number of Social Activities” was associated with increased depressive symptoms for participants living in the northern region (Table 2). Aside from cultural differences between Northern Europe and other regions, this seemingly paradoxical result aligns with some research [21,73] that suggests a natural limit to human social network size, that is, when individuals exceed this limit, social overcommitment may occur, potentially leading to emotional fatigue and poorer mental health outcomes.

Intergenerational contact has been identified as reducing loneliness in older age [25]. In this context, grandparent–grandchild interactions—such as caring for grandchildren on weekends, after school hours, or during specific occasions—are frequently associated with enhanced psychological well-being [25,74]. However, other studies have cautioned against the potential physical and psychological burdens of caregiving, particularly when the responsibility is frequent or sustained, as excessive involvement may become a source of stress rather than support [75,76]. Our findings align with this latter perspective, as we observed that individuals aged 65 to 74 and participants living in the southern and western regions who maintain regular contact with grandchildren exhibit higher levels of depressive symptoms.

The use of the internet and digital technologies has been widely recognised as a potential tool to reduce loneliness and enhance social connectedness among older adults, thereby contributing to lower levels of depressive symptoms [27,77]. In our study, initial analyses suggested that older adults who did not use the internet reported higher depressive symptoms, but after adjusting for all variables, this effect was considered insignificant. This may indicate that the protective effects of technology use are mediated by other factors, such as social support or cognitive function, rather than exerting a direct influence. Nevertheless, it is important to note that the “Use of Internet in Past 7 Days” variable measures only the internet usage over the past week, which can be reductive, as it does not distinguish between types of use or actual digital skills.

Finally, we found a connection between depressive symptoms and the “Type of Building” individuals live in but not with the “Area of Building”. Older adults who are 85 years old or more and who live in a building with three or more floors reported higher depressive symptoms (except for the ones living in the northern region) (Table 2). This finding contrasts with the existing literature [78], which typically indicates a higher rate of depression among nursing home residents compared to residents in private households. One possible explanation is that those living independently in private apartments may face greater social withdrawal despite living in the community [79]. In fact, social isolation in private homes, particularly in urban settings like apartment buildings, can go unnoticed, as neighbours often have limited interaction. Moreover, mobility constraints that hinder access to social spaces or services and environmental stressors such as noise, lack of green space and poor building conditions have been associated with worsened mental health in older populations [80].

Although this study has notable strengths, including a large sample size and its international cross-sectional design, which provides a broader perspective on the variables studied, some limitations should be addressed. First, all data from the SHARE survey are self-reported, which introduces the risk of self-report bias. Additionally, individuals who agree to participate in research studies like SHARE are often more motivated and in better health than those who decline or are unable to participate. As a result, a significant portion of older adults with comorbidities may have been underrepresented in the sample. It is also important to note that although several associations identified in this study were statistically significant, these findings should be interpreted with caution due to the large sample size. In such contexts, even very small differences or associations can reach statistical significance, without necessarily reflecting meaningful or practically relevant effects.

In conclusion, our findings confirm that late-life depression continues to disproportionately affect older women across Europe, underscoring persistent gender disparities in mental health. Depressive symptoms in older adults are not only detrimental to psychological well-being but also negatively impact physical health; thus, recognising and addressing both physical and mental health needs is essential for improving overall well-being in aged populations. Our results also highlight the importance of social integration in later life, especially to reduce loneliness, with moderate involvement in grandchild care and in social participation, which emerge as potential protective factors for mental health.

From a clinical and public health perspective, the findings of this study highlight the importance of addressing multiple sociodemographic and contextual factors in the prevention and management of depressive symptoms among older adults in Europe. These results suggest that mental health strategies should go beyond medical treatment and incorporate broader social and environmental interventions. Understanding the prevalence and key determinants of depressive symptoms is vital for designing targeted interventions and effective health policies, ultimately contributing to the sustainability of Europe’s health and social care systems in the face of demographic ageing.

## Figures and Tables

**Table 1:** Prevalence of depressive symptoms specified by age, gender and country, according to the EURO-D scale, among the 28 countries included in Wave 9 of the Survey of Health, Age and Retirement in Europe (SHARE).

	Overall (95% CI)	Overall			Male (95% CI)	Male			Female (95% CI)	Female		
		Standardised Rates (95% CI)				Standardised Rates (95% CI)				Standardised Rates (95% CI)		
		65–74 years	75–84 years	≥85 years		65–74 years	75–84 years	≥85 years		65–74 years	75–84 years	≥85 years
Austria	24.5 [24.0–25.0]	17.8 [17.3–18.4]	28.4 [27.5–29.4]	42.5 [40.7–44.3]	19.4 [18.8–20.0]	13.7 [13.0–14.4]	21.7 [20.5–22.8]	37.6 [35.3–40.1]	28.0 [27.3–28.7]	20.8 [20.0–21.7]	32.8 [31.5–34.3]	45.5 [42.9–48.2]
Belgium	30.5 [30.0–31.1]	28.5 [27.8–29.3]	31.2 [30.3–32.2]	37.1 [35.4–38.8]	21.6 [20.9–22.3]	18.8 [17.9–19.6]	23.0 [21.9–24.2]	29.8 [27.7–32.0]	37.9 [37.0–38.7]	37.3 [36.1–38.5]	37.5 [36.0–39.0]	41.3 [38.9–43.9]
Bulgaria	33.2 [32.6–33.7]	26.2 [25.5–26.9]	37.2 [36.1–38.2]	51.9 [49.9–53.9]	25.7 [25.0–26.4]	19.4 [18.5–20.2]	23.1 [21.9–24.3]	59.1 [56.1–62.2]	38.0 [37.2–38.9]	30.8 [29.8–31.9]	46.3 [44.6–48.0]	46.9 [44.2–49.6]
Croatia	32.0 [31.4–32.5]	23.7 [23.0–24.4]	36.8 [35.8–37.9]	54.1 [52.0–56.1]	23.8 [23.1–24.5]	16.7 [15.9–17.5]	26.9 [25.7–28.2]	45.6 [42.9–48.3]	38.6 [37.7–39.4]	29.9 [28.9–31.0]	44.3 [42.7–46.0]	59.8 [56.9–63.0]
Cyprus	20.9 [20.5–21.4]	15.8 [15.3–16.4]	25.1 [24.3–26.0]	31.4 [29.9–33.0]	17.9 [17.3–18.5]	16.8 [16.1–17.6]	19.2 [18.2–20.3]	18.8 [17.1–20.5]	23.4 [22.7–24.1]	15.0 [14.3–15.8]	29.8 [28.5–31.1]	42.1 [39.6–44.7]
Czech Republic	28.6 [28.0–29.1]	25.1 [24.4–25.8]	29.8 [28.8–30.7]	40.2 [38.4–42.0]	19.5 [18.9–20.2]	17.5 [16.7–18.3]	19.3 [18.3–20.4]	28.6 [26.5–30.7]	34.0 [33.1–34.8]	30.1 [29.0–31.1]	35.6 [34.2–37.1]	45.8 [43.2–48.6]
Denmark	18.4 [18.0–18.8]	17.6 [17.0–18.2]	17.2 [16.5–18.0]	24.7 [23.3–26.1]	13.4 [12.9–13.9]	12.5 [11.9–13.2]	13.5 [12.7–14.5]	16.7 [15.1–18.3]	22.4 [21.8–23.1]	21.8 [20.9–22.7]	20.8 [19.7–21.9]	29.3 [27.3–31.6]
Estonia	37.3 [36.7–38.0]	32.0 [31.2–32.8]	41.4 [40.3–42.5]	49.3 [47.4–51.3]	29.6 [28.8–30.4]	25.0 [24.1–26.0]	32.0 [30.6–33.4]	42.7 [40.2–45.4]	41.5 [40.6–42.4]	36.5 [35.4–37.7]	45.6 [44.0–47.3]	51.8 [49.0–54.7]
Finland	26.4 [25.9–27.0]	21.6 [21.0–22.3]	29.9 [29.0–30.9]	37.5 [35.8–39.2]	20.7 [20.0–21.3]	15.4 [14.6–16.1]	24.1 [22.9–25.3]	34.1 [31.8–36.5]	31.5 [30.7–32.3]	27.3 [26.3–28.3]	35.1 [33.6–36.5]	39.7 [37.3–42.3]
France	33.5 [33.0–34.1]	28.3 [27.6–29.0]	37.3 [36.3–38.4]	45.5 [43.7–47.4]	26.0 [25.3–26.7]	23.7 [22.8–24.7]	26.0 [24.7–27.2]	35.8 [33.5–38.2]	39.2 [38.3–40.1]	32.3 [31.2–33.4]	45.9 [44.2–47.5]	50.9 [48.1–53.7]
Germany	25.2 [24.7–25.7]	21.3 [20.7–21.9]	27.5 [26.6–28.4]	35.9 [34.3–37.6]	19.0 [18.4–19.6]	16.4 [15.7–17.2]	20.7 [19.6–21.9]	25.0 [23.1–27.0]	31.1 [30.4–31.9]	25.6 [24.7–26.6]	34.0 [32.6–35.4]	47.0 [44.4–49.8]
Greece	20.2 [19.8–20.7]	12.2 [11.7–12.6]	22.6 [21.8–23.5]	47.7 [45.8–49.7]	14.7 [14.2–15.3]	8.4 [7.9–9.0]	14.5 [13.6–15.4]	41.7 [39.2–44.4]	24.9 [24.2–25.6]	15.2 [14.4–15.9]	30.0 [28.7–31.4]	52.4 [49.6–55.3]
Hungary	28.9 [28.3–29.4]	20.0 [19.4–20.6]	35.1 [34.0–36.1]	50.0 [48.1–52.0]	23.6 [22.9–24.3]	17.1 [16.3–17.9]	28.5 [27.2–29.8]	38.1 [35.7–40.6]	32.3 [31.5–33.1]	22.2 [21.4–23.2]	39.7 [38.2–41.3]	55.6 [52.7–58.6]
Israel	33.8 [33.2–34.3]	25.7 [25.0–26.4]	34.5 [33.5–35.5]	65.9 [63.7–68.2]	24.4 [23.7–25.1]	16.3 [15.6–17.1]	21.6 [20.5–22.8]	65.9 [62.8–69.2]	40.1 [39.2–41.0]	31.3 [30.2–32.4]	44.3 [42.7–46.0]	65.9 [62.8–69.2]
Italy	34.3 [33.7–34.9]	27.3 [26.6–28.0]	38.7 [37.6–39.8]	52.4 [50.4–54.4]	26.6 [25.9–27.3]	17.8 [17.0–18.6]	32.5 [31.1–33.9]	48.1 [45.5–50.9]	40.5 [39.6–41.4]	34.9 [33.7–36.0]	43.9 [42.3–45.6]	55.6 [52.7–58.6]
Latvia	28.0 [27.5–28.5]	24.0 [23.3–24.7]	28.0 [27.1–28.9]	44.6 [42.8–46.5]	21.5 [20.8–22.1]	17.8 [17.0–18.6]	22.9 [21.7–24.0]	33.3 [31.1–35.7]	31.7 [30.9–32.5]	28.3 [27.3–29.3]	30.3 [28.9–31.6]	49.4 [46.7–52.2]
Lithuania	42.6 [42.0–43.3]	36.6 [35.8–37.4]	45.7 [44.6–46.9]	60.0 [57.9–62.2]	33.0 [32.2–33.9]	29.1 [28.0–30.1]	34.0 [32.6–35.4]	47.2 [44.6–50.0]	47.7 [46.8–48.7]	41.4 [40.2–42.7]	51.5 [49.7–53.2]	64.4 [61.3–67.6]
Luxembourg	28.1 [27.6–28.7]	29.1 [28.4–29.9]	27.2 [26.3–28.1]	26.5 [25.1–27.9]	23.5 [22.9–24.2]	23.3 [22.4–24.2]	22.3 [21.2–23.5]	27.8 [25.8–29.9]	32.3 [31.5–33.1]	34.0 [32.9–35.2]	32.2 [30.9–33.6]	25.0 [23.1–27.0]
Malta	37.2 [36.5–37.8]	31.7 [30.9–32.4]	41.4 [40.3–42.6]	49.1 [47.2–51.1]	25.7 [25.0–26.4]	22.3 [21.4–23.2]	30.0 [28.7–31.4]	28.6 [26.5–30.7]	46.2 [45.3–47.2]	40.7 [39.4–41.9]	49.2 [47.6–51.0]	61.8 [58.7–64.9]
Netherlands	19.1 [18.7–19.6]	17.1 [16.5–17.6]	21.9 [21.1–22.8]	20.4 [19.2–21.7]	14.5 [14.0–15.1]	12.0 [11.4–12.7]	18.5 [17.5–19.6]	14.7 [13.2–16.3]	23.1 [22.4–23.8]	21.3 [20.5–22.2]	25.2 [23.9–26.4]	25.0 [23.1–27.0]
Poland	36.4 [35.8–37.0]	28.6 [27.9–29.4]	40.9 [39.8–42.0]	57.8 [55.7–60.0]	26.3 [25.6–27.0]	20.3 [19.4–21.1]	30.9 [29.6–32.3]	39.4 [37.0–42.0]	44.3 [43.4–45.2]	35.5 [34.3–36.6]	47.9 [46.2–49.6]	72.2 [68.9–75.6]
Portugal	44.5 [43.9–45.2]	40.2 [39.4–41.1]	48.6 [47.4–49.8]	51.8 [49.8–53.8]	28.3 [27.5–29.0]	26.0 [25.0–27.0]	28.6 [27.3–29.9]	37.0 [34.7–39.5]	57.9 [56.9–59.0]	52.6 [51.2–54.0]	63.6 [61.7–65.6]	65.5 [62.4–68.8]
Romania	38.6 [38.0–39.2]	31.9 [31.1–32.7]	41.0 [39.9–42.2]	60.2 [58.1–62.4]	28.8 [28.1–29.6]	23.2 [22.3–24.1]	32.3 [30.9–33.7]	43.3 [40.8–46.0]	46.1 [45.1–47.0]	39.3 [38.1–40.5]	47.9 [46.2–49.6]	69.8 [66.6–73.2]
Slovakia	26.4 [25.9–26.9]	19.9 [19.3–20.5]	29.5 [28.6–30.5]	45.5 [43.6–47.4]	21.5 [20.9–22.2]	15.2 [14.5–16.0]	25.5 [24.2–26.7]	37.5 [35.1–40.0]	30.6 [29.8–31.4]	24.8 [23.8–25.7]	32.5 [31.1–33.9]	50.0 [47.3–52.9]
Slovenia	25.2 [24.7–25.7]	20.7 [20.1–21.3]	27.3 [26.4–28.2]	38.7 [37.0–40.5]	17.3 [16.7–17.9]	14.1 [13.4–14.9]	18.2 [17.2–19.2]	28.5 [26.4–30.6]	31.0 [30.2–31.8]	25.8 [24.9–26.8]	34.0 [32.5–35.4]	44.9 [42.3–47.6]
Spain	34.5 [33.9–35.1]	26.7 [26.0–27.4]	40.8 [39.7–41.9]	50.8 [48.8–52.8]	23.7 [23.0–24.4]	17.7 [16.9–18.5]	27.6 [26.4–28.9]	38.5 [36.1–41.0]	42.9 [41.9–43.8]	33.8 [32.6–34.9]	51.1 [49.4–52.9]	59.6 [56.6–62.7]
Sweden	23.2 [22.8–23.7]	20.7 [20.1–21.4]	23.5 [22.7–24.3]	33.2 [31.6–34.8]	16.5 [16.0–17.1]	13.2 [12.5–13.9]	18.1 [17.0–19.1]	26.5 [24.6–28.6]	28.9 [28.1–29.6]	26.8 [25.8–27.8]	28.4 [27.1–29.7]	38.9 [36.5–41.5]
Switzerland	19.1 [18.6–19.5]	17.4 [16.9–18.0]	20.0 [19.2–20.7]	23.7 [22.4–25.1]	11.9 [11.4–12.4]	10.4 [9.8–11.0]	13.2 [12.4–14.1]	14.9 [13.4–16.5]	25.0 [24.3–25.7]	23.1 [22.1–24.0]	26.4 [25.2–27.7]	29.3 [27.2–31.5]
Total	29.2 [28.7–29.8]	24.2 [23.5–24.8]	31.9 [30.9–32.9]	43.6 [41.8–45.5]	21.5 [20.9–22.2]	17.3 [16.6–18.2]	23.2 [22.1–24.4]	34.7 [32.4–37.1]	35.1 [34.2–35.9]	29.6 [28.6–30.7]	38.3 [36.8–39.9]	49.4 [46.7–52.2]

CI—confidence interval.

**Table 2:** Association of explanatory variables with depressive symptoms (unadjusted and adjusted models).

		*N*	*N* (%) Depressive Symptoms	Unadjusted Model			Adjusted Model		
		38,092	10,815 (28.4)	OR	CI 95	*p*	OR	CI 95	*p*
Sociodemographics	Gender								
	Male	16,254	3301 (8.7)	1	–	–	1	–	–
	Female	21,838	7514 (19.7)	2.046	1.950–2.146	<0.001	1.980	1.862–2.106	<0.001
	Age								
	65–74 years	20,571	4925 (12.9)	1	–	–	1	–	–
	75–84 years	13,577	4262 (11.2)	1.488	1.417–1.563	<0.001	0.956	0.898–1.018	0.163
	≥85 years	3944	1628 (4.3)	2.274	2.115–2.445	<0.001	0.834	0.754–0.922	<0.001
	Marital Status								
	Never married	1590	427 (1.1)	1	–	–	1	–	–
	Married or registered partnership	24,858	6217 (16.3)	0.902	0.803–1.013	0.081	1.188	1.025–1.378	0.022
	Divorced	3058	907 (2.4)	1.176	1.025–1.349	0.020	1.017	0.859–1.205	0.842
	Widowed	8586	3264 (8.6)	1.628	1.443–1.837	<0.001	1.107	0.949–1.290	0.196
	Years of Education								
	Low education (0–8)	10,524	3663 (9.7)	1	–	–	1	–	–
	Moderate education (9–12)	14,736	4074 (10.7)	0.692	0.653–0.734	<0.001	1.048	0.975–1.127	0.201
	High education (13–16)	9091	2299 (6.0)	0.608	0.568–0.650	<0.001	1.134	1.043–1.233	0.003
	Very high education (17+)	3741	779 (2.0)	0.478	0.435–0.524	<0.001	1.079	0.964–1.207	0.188
	Shortage of Money								
	Never	14,659	3444 (9.0)	1	–	–	1	–	–
	Rarely	8733	2160 (5.7)	1.045	0.982–1.113	0.167	0.681	0.631–0.735	<0.001
	Sometimes	9197	2802 (7.4)	1.406	1.322–1.495	<0.001	0.653	0.604–0.705	<0.001
	Often	5503	2409 (6.3)	2.716	2.527–2.920	<0.001	0.718	0.653–0.790	<0.001
	Job Situation								
	Retired	33,481	9343 (24.5)	1	–	–	–	–	–
	Employed	1425	234 (0.6)	0.486	0.420–0.562	<0.001	–	–	–
	Unemployed and others	3186	1238 (3.3)	1.746	1.608–1.895	<0.001	–	–	–
Physical Health	Number of Chronic Diseases								
	0	5709	742 (1.9)	1	–	–	1	–	–
	1	9274	1963 (5.2)	1.787	1.629–1.960	<0.001	1.127	1.016–1.251	0.024
	2 or more	23,109	8110 (21.3)	3.621	3.334–3.933	<0.001	1.276	1.157–1.407	<0.001
	Number of Limitations Performing Activities of Daily Living								
	0	33,828	8335 (21.9)	1	–	–	1	–	–
	1 or more	4264	2480 (6.5)	4.220	3.948–4.511	<0.001	1.206	1.103–1.318	<0.001
	Number of Limitations Performing Instrumental Activities of Daily Living								
	0	30,328	6593 (17.3)	1	–	–	1	–	–
	1 or more	7764	4222 (11.1)	4.425	4.194–4.668	<0.001	1.325	1.230–1.428	<0.001
	Limitation in Activities Because of Health								
	Severely limited	6397	3587 (9.4)	1	–	–	1	–	–
	Limited, but not severely	13,493	4513 (11.8)	0.386	0.363–0.411	<0.001	0.783	0.725–0.846	<0.001
	Not limited	18,202	2715 (7.2)	0.135	0.126–0.144	<0.001	0.559	0.511–0.611	<0.001
	Hearing								
	Good, very good or excellent	29,850	7461 (19.6)	1	–	–	1	–	–
	Fair or poor	8242	3354 (8.8)	1.987	1.886–2.093	<0.001	1.233	1.155–1.317	<0.001
	Vision								
	Good, very good or excellent	29,196	6889 (18.1)	1	–	–	1	–	–
	Fair or poor	8896	3926 (10.3)	2.430	2.307–2.559	<0.001	1.211	1.136–1.292	<0.001
	Pain								
	No	19,681	3434 (9.0)	1	–	–	1	–	–
	Mild	3888	973 (2.5)	1.526	1.405–1.657	<0.001	1.282	1.168–1.406	<0.001
	Moderate	10,647	4177 (11.0)	2.961	2.804–3.127	<0.001	1.503	1.407–1.606	<0.001
	Severe	3876	2231 (5.9)	6.219	5.772–6.700	<0.001	2.040	1.858–2.240	<0.001
	Stayed Overnight in Hospital for the Last 12 Months								
	No	32,611	8566 (22.5)	1	–	–	1	–	–
	Yes	5481	2249 (5.9)	1.994	1.877–2.119	<0.001	1.344	1.248–1.447	<0.001
	Received Professional Health Services								
	No	34,271	8967 (23.5)	1	–	–	1	–	–
	Yes	3821	1848 (4.9)	2.953	2.751–3.170	<0.001	1.099	1.004–1.204	0.042
Mental Health	Quality of Life and Well-Being								
	CASP scale	38,092	10,815 (28.4)	0.818	0.814–0.822	<0.001	0.864	0.859–0.870	<0.001
	Loneliness								
	Without signs of loneliness	31,577	7003 (18.4)	1	–	–	1	–	–
	With signs of loneliness	6515	3812 (10.0)	5.325	5.022–5.646	<0.001	2.111	1.967–2.267	<0.001
Behavioural Factors	Physical Inactivity								
	Engage in vigorous or moderate physical activity	32,701	8039 (21.1)	1	–	–	–	–	–
	Never engage in vigorous nor moderate physical activity	5391	2776 (7.3)	3.157	2.970–3.356	<0.001	–	–	–
	Ever Smoked Daily								
	Yes	14,588	3853 (10.1)	1	–	–	1	–	–
	No	23,504	6962 (18.3)	1.109	1.058–1.163	<0.001	0.890	0.839–0.945	<0.001
	Alcohol Intake								
	Yes	17,519	3914 (10.3)	1	–	–	–	–	–
	No	20,573	6901 (18.1)	1.673	1.593–1.758	<0.001	–	–	–
Social Participation	Number of Social Activities								
	No activities	8054	3163 (8.3)	1	–	–	1	–	–
	One activity	9147	2742 (7.2)	0.619	0.579–0.662	<0.001	1.123	1.030–1.225	0.009
	Two or more activities	20,891	4910 (12.9)	0.434	0.406–0.464	<0.001	1.112	1.015–1.218	0.022
	Satisfaction with Social Activities								
	Satisfaction with activities	38,092	10,815 (28.4)	0.796	0.787–0.804	<0.001	0.933	0.920–0.946	<0.001
	Network Satisfaction								
	Network satisfaction	38,092	10,815 (28.4)	0.841	0.827–0.855	<0.001	0.975	0.955–0.995	0.016
	Looking after grandchildren								
	Without grandchildren	5407	1459 (3.8)	1	–	–	1	–	–
	With grandchildren but do not look after them	22,282	6990 (18.4)	1.182	1.105–1.265	<0.001	1.015	0.930–1.108	0.736
	With grandchildren and looking after them	10,403	2366 (6.2)	0.786	0.728–0.849	<0.001	1.143	1.038–1.260	0.007
Internet Skills	Use of Internet in Past 7 Days								
	Yes	21,103	4721 (12.4)	1	–	–	–	–	–
	No	16,989	6094 (16.0)	1.929	1.836–2.025	<0.001	–	–	–
Living conditions	Area of Building								
	A big city, the suburbs or outskirts of a big city	9357	2573 (6.8)	1	–	–	–	–	–
	A large town	6346	1847 (4.8)	1.027	0.955–1.105	0.468	–	–	–
	A small town	9415	2664 (7.0)	0.974	0.911–1.041	0.440	–	–	–
	A rural area or village	12,974	3731 (9.8)	1.010	0.949–1.075	0.746	–	–	–
	Type of Building								
	A farmhouse	2579	792 (2.1)	1	–	–	1	–	–
	A freestanding one/two-family house or a one- or two-family house as a row or double house	22,655	6086 (16.0)	0.315	0.287–0.345	<0.001	1.059	0.950–1.182	0.301
	A building with 3 or more floors	12,744	3890 (10.2)	0.363	0.330–0.399	<0.001	1.136	1.014–1.273	0.027
	A housing complex with services for older people or a nursing home	114	47 (0.1)	0.741	0.502–1.093	0.130	1.107	0.693–1.769	0.671

CI—confidence interval; OR—odds ratios.

## Data Availability

The data presented in this study are openly available in http://www.share-project.org/ (accessed on 27 February 2025) (SHARE Wave 9—https://doi.org/10.6103/SHARE.w9.900).

## Supplementary Materials

The following supporting information can be downloaded at: https://www.mdpi.com/article/10.3390/jcm14155340/s1, Supplementary Materials and Methods; Table S1: Association between explanatory variables and depressive symptoms by age group; Table S2: Association between explanatory variables and depressive symptoms by European regions.

## Abbreviations

The following abbreviations are used in this manuscript:

SHARE	Survey of Health, Ageing and Retirement in Europe
ADLs	Activities of Daily Living
iADLs	Instrumental Activities of Daily Living
