# Supplementary material for: Prevalence and Determinants of Depressive Symptoms in Older Adults Across Europe: Evidence from SHARE Wave 9
Source: J Clin Med. 2025 Jul 29;14(15):5340. doi: 10.3390/jcm14155340 (PMC12348025; doi:10.3390/jcm14155340)
Supplement: Supplementary file 1 [file jcm-14-05340-s001.zip › Supplementary Materials.pdf]

Supplementary Materials

EURO-D scale

Depressive symptoms were evaluated using the EURO-D scale that measures current levels of depression based on the following questions: “In the last month, have you been sad or depressed?” (depression); “What are your hopes for the future?” (pessimism); “In the last month, have you felt that you would rather be dead?” (suicidality); “Do you tend to blame yourself or feel guilty about anything?” (guilt); “Have you had trouble sleeping recently?” (sleep); “In the last month, what is your interest in things?” (interest); “Have you been irritable recently?” (irritability); “What has your appetite been like?” (appetite); “In the last month, have you had too little energy to do the things you wanted to do?” (fatigue); “How is your concentration? For example, can you concentrate on a television program, film, or radio program?” and “Can you concentrate on something you read?” (concentration); “What have you enjoyed doing recently? (enjoyment); “In the last month, have you cried at all?” (tearfulness). Every item was scored 1 if present, and 0 if absent and the final scores ranged from 0 ("not depressed") to 12 ("highly depressed") and were dichotomized into "Without depressive symptoms" (scores <4) and "With depressive symptoms" (scores ≥4)

Sociodemographic variables

Marital status was evaluated through the question, "What is your marital status?", with responses recoded into four categories: "Never married," "Married or in a registered partnership," "Divorced," and "Widowed."

Years of education was assessed using the question, "How many years have you been in full-time education?", which included receiving tuition, engaging in practical work or supervised study or taking exams. Responses were grouped into four levels: "Low education (0–8 years)," "Moderate education (9–12 years)," "High education (13–16 years)," and "Very high education (17 or more years)."

The question "How often do you think that shortage of money stops you from doing the things you want to do?" determined the shortage of money, with the response options: "Often," "Sometimes," "Rarely," and "Never."

Job situation was identified with the question, “In general, which of the following best describes your current employment situation?” and the responses were categorised into three groups: “Retired”, “Employed” (including self-employed and working for family business) and “Unemployed and others” (including permanently sick/disabled, homemaker or other).

Physical Health variables

The number of chronic diseases was determined based on self-reported diagnoses and grouped into three levels: “No chronic diseases”, “One chronic disease” and “Two or more chronic diseases”.

The number of Limitations in Activities of Daily Living (ADLs) was calculated by summing reported difficulties in six tasks: dressing (including shoes and socks), walking across a room, bathing or showering, eating (such as cutting food), getting in or out of bed, and using the toilet (including getting up or down). The number of Limitations in Instrumental Activities of Daily Living (iADLs) was determined from the total difficulties in tasks such as using a map in an unfamiliar place, preparing a hot meal, grocery shopping, making telephone calls, taking medications, performing housework or gardening, managing money, leaving the house independently or accessing transportation, and doing personal laundry. Both variables were categorised into "No limitations” and "One limitation or more".

Limitation in activities because of health was assessed using the question "For the past six months at least, to what extent have you been limited because of a health problem in activities people usually do?" with responses grouped as "Severely limited," "Limited, but not severely," and "Not limited."

Hearing was evaluated with the question “Is your hearing (using a hearing aid as usual)”. Vision was assessed using the questions “How good is your eyesight for seeing things at a distance, like recognizing a friend across the street (using glasses or contact lenses as usual)? and “How good is your eyesight for seeing things up close, like reading ordinary newspaper print (using glasses or contact lenses as usual)?”. Responses of both variables were classified into two levels: “Good, very good or excellent” and “Fair or poor”.

Pain was established through two questions: “Are you troubled with pain?” and “How bad is the pain most of the time? Is it...”. The responses were categorised into “No Pain”, “Mild pain”, “Moderate pain” and “Severe pain”.

Hospital Stays in the Last 12 Months were evaluated with the question "During the last twelve months, have you been in a hospital overnight? Please consider stays in medical, surgical, psychiatric, or any other specialized wards," with responses recorded as "Yes" or "No."

Received professional health services was identified by the question “During the last twelve months, did you receive in your own home any professional or paid services listed on this card due to a physical, mental, emotional or memory problem? Help with personal care, (e.g. getting in and out of bed, dressing, bathing and showering); Help with domestic tasks (e.g. cleaning, ironing, cooking); Meals-on-wheels (i.e. ready-made

meals provided by a municipality or a private provider); Help with other activities (e.g. filling in a drug dispenser); None of the above. Responses were recorded as “Yes” or “No”.

Mental Health variables

Quality of Life and Well-Being were measured through the Control, Autonomy, Self-realization, and Pleasure (CASP-12) scale, a 12-item self-assessment questionnaire composed by the following questions: “How often do you think your age prevents you from doing the things you would like to do?”, “How often do you feel that what happens to you is out of your control?”, “How often do you feel left out of things?”, “How often do you think that you can do the things that you want to do?”, “How often do you think that family responsibilities prevent you from doing what you want to do?”, “How often do you think that shortage of money stops you from doing the things you want to do?”, “How often do you look forward to each day?”, “How often do you feel that your life has meaning?”, “How often, on balance, do you look back on your life with a sense of happiness?”, “How often do you feel full of energy these days?”, “How often do you feel that life is full of opportunities?” and “How often do you feel that the future looks good for you?”. Each question is rated from 1 (never) to 4 (often) and the total score ranges from 12 to 48, with higher scores meaning better quality of life.

Loneliness was evaluated by the Three-Item Loneliness Scale that measures indirect loneliness and is composed of the following questions: “How much of the time do you feel you lack companionship?”; “How much of the time do you feel left out?” and “How much of the time do you feel isolated from others?”. The three items companionship, left out, and isolated are answered on a three-point Likert scale (“often”, “some of the time”, “hardly ever or never”) and the total scores range from 3 to 9, with higher scores indicating greater loneliness. We grouped the final answers into two categories: “Not lonely” for scores between 3 and 5 and “Lonely” for scores of 6 or higher.

Behavioural Factors variables

Physical inactivity was evaluated through two questions “How often do you engage in vigorous physical activity, such as sports, heavy housework, or a job involving physical labour?" and "How often do you engage in activities that require a moderate level of energy, such as gardening, cleaning the car, or going for a walk?". For both, the response options were: "More than once a week", "Once a week", "One to three times a month" and "Hardly ever or never”. This variable was recoded into two groups: “Engage in

vigorous or moderate physical activity” and “Never engage in vigorous or moderate physical activity”.

Ever smoked daily was assessed by the question, "Have you ever smoked cigarettes, cigars, cigarillos, or a pipe daily for at least one year?" with possible responses being "Yes" or "No."

Alcohol intake was established by asking "During the last 7 days, have you had at least one alcoholic beverage?" with the response options "Yes" or "No."

Social Participation variables

Number of social activities was assessed by asking participants “Which of the activities listed, if any, have you done in the last twelve months? Done voluntary or charity work; Attended an educational or training course; Gone to a sport, social or other kind of club; Taken part in a political or community-related organization; Read books, magazines or newspapers; Did word or number games such as crossword puzzles or Sudoku; Played cards or games such as chess; None of these”. Answers were grouped into three categories: “No activities”, “One activity” and “Two or more activities”.

Satisfaction with social activities was evaluated through the question “On a scale from 0 to 10 where 0 means completely dissatisfied and 10 means completely satisfied, how satisfied are you with the activities that you mentioned?”.

Network satisfaction was established through the question: “Overall, how satisfied are you with the relationship that you have with the person/relationships that you have with the persons we have just talked about? Please answer on a scale from 0 to 10, where 0 means completely dissatisfied, and 10 means completely satisfied.”

Looking after grandchildren was assessed using two questions: “Talking about grandchildren, how many grandchildren do you (and your husband/ wife/ partner) have altogether? (Include grandchildren from previous relationships)” and “During the last twelve months, have you regularly or occasionally looked after your grandchild/ your grandchildren without the presence of the parents?”. The responses were grouped into three possibilities “Without grandchildren”, “With grandchildren but don’t look after them” and “With grandchildren and looking after them”.

Internet skills variables

Internet use was determined by asking the participants “During the past 7 days, have you used the Internet, for e-mailing, searching for information, making purchases, or for any other purpose at least once? Any other purpose includes chatting, social networks, skyping etc.”. Responses were recorded as “Yes” and “No”.

Living conditions variables

Area of Building was assessed through the question, "How would you describe the area where you live?" Responses were recoded into four categories: "A big city, the suburbs or outskirts of a big city," "A large town", “A small town” and "A rural area or village”.

Type of Building was identified by asking "Which type of building does the household live in?" Responses were grouped into four categories: "A farmhouse", "A free-standing one- or two-family house or a one- or two-family house as a row or double house", "A building with three or more floors," and "A housing complex with services for older adults or a nursing home”.
