# Supplementary material for: Prevalence and Determinants of Depressive Symptoms in Older Adults Across Europe: Evidence from SHARE Wave 9
Source: J Clin Med. 2025 Jul 29;14(15):5340. doi: 10.3390/jcm14155340 (PMC12348025; doi:10.3390/jcm14155340)
Supplement: Supplementary file 1 [file jcm-14-05340-s001.zip › Table S1.pdf]

Table S1 - Association between explanatory variables and depressive symptoms by age group.

				C.I. 95%	
**Age groups**	**Variables**	***p***	**O.R.**	**Lower**	**Upper**
**65-74**	Male or female (1)	0.000	2.129	1.951	2.323
	Marital status	0.586			
	Marital status (1)	0.992	1.001	0.830	1.207
	Marital status (2)	0.964	1.005	0.813	1.243
	Marital status (3)	0.459	1.080	0.881	1.323
	Years of Education	0.915			
	Years of Education (1)	0.887	1.007	0.910	1.116
	Years of Education (2)	0.600	1.032	0.917	1.162
	Years of Education (3)	0.873	0.987	0.842	1.157
	Shortage of money stops	0.000			
	Shortage of money stops (1)	0.000	0.681	0.612	0.759
	Shortage of money stops (2)	0.000	0.629	0.564	0.701
	Shortage of money stops (3)	0.000	0.666	0.585	0.758
	Current job situation	0.573			
	Current job situation (1)	0.397	1.077	0.907	1.279
	Current job situation (2)	0.558	0.961	0.840	1.099
	Number of chronic diseases	0.007			
	Number of chronic diseases (1)	0.030	1.157	1.014	1.319
	Number of chronic diseases (2)	0.002	1.220	1.077	1.381
	Number of limitations with activities of daily living (ADL) (1)	0.004	1.240	1.073	1.433
	Limitations with instrumental activities of daily living (iadl) (1)	0.000	1.282	1.140	1.442
	Limited in activities because of health	0.000			
	Limited in activities because of health (1)	0.000	0.774	0.689	0.871
	Limited in activities because of health (2)	0.000	0.501	0.441	0.570
	Hearing (1)	0.000	1.273	1.151	1.408
	Vision (1)	0.000	1.263	1.151	1.385
	Pain and level of pain	0.000			
	Pain and level of pain (1)	0.000	1.329	1.173	1.506
	Pain and level of pain (2)	0.000	1.611	1.469	1.767
	Pain and level of pain (3)	0.000	2.116	1.848	2.422
	Network satisfaction	0.024	0.967	0.939	0.996
	CASP index	0.000	0.871	0.863	0.879
	Looks after grandchildren	0.001			
	Looks after grandchildren (1)	0.464	1.045	0.929	1.175
	Looks after grandchildren (2)	0.003	1.202	1.065	1.357
	Loneliness (1)	0.000	2.030	1.832	2.249
	Number of activities in last year	0.005			
	Number of activities in last year (1)	0.071	1.118	0.990	1.263
	Number of activities in last year (2)	0.001	1.220	1.082	1.376
	Satisfaction with activities	0.000	0.945	0.926	0.965
	Ever smoked daily (1)	0.000	0.830	0.766	0.898
	At least one alcoholic beverage the last 7 days (1)	0.107	0.934	0.860	1.015
	Physical inactivity (1)	0.981	1.002	0.881	1.139
	Stayed overnight in hospital last 12 months (1)	0.000	1.474	1.323	1.642
	Received professional services (1)	0.063	1.168	0.992	1.376

	Use of internet in past 7 days (1)	0.007	0.882	0.804	0.967
	Area of building	0.912			
	Area of building (1)	0.537	0.968	0.873	1.074
	Area of building (2)	0.818	0.986	0.874	1.112
	Area of building (3)	0.552	0.966	0.863	1.082
	Type of building	0.713			
	Type of building (1)	0.614	1.040	0.894	1.210
	Type of building (2)	0.887	0.988	0.834	1.170
	Type of building (3)	0.988	1.010	0.300	3.395
**75-84**	Male or female (1)	0.000	1.946	1.755	2.159
	Marital status	0.007			
	Marital status (1)	0.061	1.292	0.989	1.687
	Marital status (2)	0.988	1.002	0.739	1.359
	Marital status (3)	0.325	1.145	0.874	1.501
	Years of Education	0.038			
	Years of Education (1)	0.743	1.018	0.913	1.135
	Years of Education (2)	0.010	1.187	1.042	1.352
	Years of Education (3)	0.187	1.129	0.943	1.352
	Shortage of money stops	0.000			
	Shortage of money stops (1)	0.000	0.677	0.600	0.764
	Shortage of money stops (2)	0.000	0.626	0.553	0.707
	Shortage of money stops (3)	0.000	0.555	0.477	0.647
	Current job situation	0.694			
	Current job situation (1)	0.555	1.163	0.705	1.919
	Current job situation (2)	0.541	0.952	0.812	1.116
	Number of chronic diseases	0.258			
	Number of chronic diseases (1)	0.625	0.955	0.793	1.150
	Number of chronic diseases (2)	0.591	1.049	0.882	1.247
	Number of limitations with activities of daily living (ADL) (1)	0.003	1.231	1.074	1.411
	Limitations with instrumental activities of daily living (iADL)(1)	0.000	1.275	1.141	1.425
	Limited in activities because of health	0.000			
	Limited in activities because of health (1)	0.000	0.742	0.659	0.836
	Limited in activities because of health (2)	0.000	0.501	0.436	0.576
	Hearing (1)	0.000	1.345	1.219	1.484
	Vision (1)	0.000	1.325	1.199	1.464
	Pain and level of pain	0.000			
	Pain and level of pain (1)	0.002	1.268	1.090	1.475
	Pain and level of pain (2)	0.000	1.360	1.225	1.509
	Pain and level of pain (3)	0.000	2.060	1.777	2.388
	Network satisfaction	0.004	0.954	0.924	0.985
	CASP index for quality of life and well-being	0.000	0.880	0.871	0.890
	Looks after grandchildren	0.083			
	Looks after grandchildren (1)	0.974	0.998	0.862	1.155
	Looks after grandchildren (2)	0.136	1.142	0.959	1.360
	Loneliness (1)	0.000	1.972	1.766	2.203
	Number of activities in last year	0.001			
	Number of activities in last year (1)	0.005	1.217	1.060	1.396
	Number of activities in last year (2)	0.000	1.300	1.127	1.498
	Satisfaction with activities	0.000	0.936	0.915	0.958
	Ever smoked daily (1)	0.836	0.990	0.897	1.092
	At least one alcoholic beverage the last 7 days (1)	0.103	0.922	0.837	1.016

	Physical inactivity (1)	0.136	1.099	0.971	1.245
	Stayed overnight in hospital last 12 months (1)	0.000	1.336	1.192	1.497
	Received professional services (1)	0.000	1.279	1.123	1.456
	Use of internet in past 7 days (1)	0.465	1.040	0.937	1.154
	Area of building	0.060			
	Area of building (1)	0.632	1.030	0.913	1.162
	Area of building (2)	0.724	0.975	0.847	1.123
	Area of building (3)	0.041	0.872	0.764	0.994
	Type of building	0.292			
	Type of building (1)	0.471	1.069	0.891	1.282
	Type of building (2)	0.132	1.167	0.954	1.427
	Type of building (3)	0.808	0.907	0.413	1.992
**85+**	Male or female (1)	0.000	1.753	1.435	2.141
	Marital status	0.186			
	Marital status (1)	0.819	1.063	0.633	1.785
	Marital status (2)	0.979	1.008	0.545	1.865
	Marital status (3)	0.588	0.870	0.525	1.440
	Years of Education	0.589			
	Years of Education (1)	0.296	1.108	0.914	1.342
	Years of Education (2)	0.226	1.160	0.912	1.476
	Years of Education (3)	0.463	1.138	0.806	1.605
	Shortage of money stops	0.000			
	Shortage of money stops (1)	0.000	0.645	0.519	0.800
	Shortage of money stops (2)	0.000	0.566	0.453	0.708
	Shortage of money stops (3)	0.000	0.480	0.362	0.636
	Current job situation	0.644			
	Current job situation (1)	0.502	0.417	0.033	5.347
	Current job situation (2)	0.510	0.917	0.709	1.186
	Number of chronic diseases	0.010			
	Number of chronic diseases (1)	0.363	1.184	0.823	1.705
	Number of chronic diseases (2)	0.020	1.499	1.065	2.109
	Number of limitations with activities of daily living (ADL) (1)	0.008	1.313	1.074	1.604
	Limitations with instrumental activities of daily living (iADL)(1)	0.445	1.075	0.893	1.295
	Limited in activities because of health	0.000			
	Limited in activities because of health (1)	0.002	0.734	0.605	0.890
	Limited in activities because of health (2)	0.000	0.571	0.447	0.731
	Hearing (1)	0.000	1.381	1.174	1.625
	Vision (1)	0.168	1.127	0.951	1.336
	Pain and level of pain	0.000			
	Pain and level of pain (1)	0.496	1.103	0.832	1.462
	Pain and level of pain (2)	0.000	1.453	1.202	1.756
	Pain and level of pain (3)	0.000	2.251	1.760	2.878
	Network satisfaction	0.229	0.966	0.914	1.022
	CASP index	0.000	0.878	0.861	0.894
	Looks after grandchildren	0.159			
	Looks after grandchildren (1)	0.338	1.142	0.870	1.500
	Looks after grandchildren (2)	0.055	1.577	0.989	2.513
	Loneliness (1)	0.000	1.891	1.576	2.269
	Number of activities in last year	0.080			
	Number of activities in last year (1)	0.075	1.229	0.979	1.544
	Number of activities in last year (2)	0.920	1.013	0.793	1.293

	Satisfaction with activities	0.000	0.923	0.890	0.957
	Ever smoked daily (1)	0.218	0.881	0.721	1.078
	At least one alcoholic beverage the last 7 days (1)	0.854	0.983	0.816	1.183
	Physical inactivity (1)	0.006	1.295	1.077	1.557
	Stayed overnight in hospital last 12 months (1)	0.014	1.294	1.053	1.589
	Received professional services (1)	0.983	0.998	0.832	1.197
	Use of internet in past 7 days (1)	0.323	1.117	0.897	1.392
	Area of building	0.000			
	Area of building (1)	0.241	0.878	0.705	1.092
	Area of building (2)	0.002	0.664	0.513	0.858
	Area of building (3)	0.000	0.634	0.499	0.806
	Type of building	0.000			
	Type of building (1)	0.308	1.175	0.862	1.602
	Type of building (2)	0.003	1.690	1.197	2.387
	Type of building (3)	0.043	2.072	1.024	4.196

C.I., confidence interval; O.R., odd ratios
