# Supplementary material for: Prevalence and Determinants of Depressive Symptoms in Older Adults Across Europe: Evidence from SHARE Wave 9
Source: J Clin Med. 2025 Jul 29;14(15):5340. doi: 10.3390/jcm14155340 (PMC12348025; doi:10.3390/jcm14155340)
Supplement: Supplementary file 1 [file jcm-14-05340-s001.zip › Table S2.pdf]

Table S2 - Association between explanatory variables and depressive symptoms by European regions.

European regions	Variables	*p*	O.R.	C.I. 95% Lower	C.I. 95% Upper
**Northern**	Male or female (1)	0.000	2.208	1.926	2.530
	Marital status	0.215			
	Marital status (1)	0.104	1.260	0.954	1.665
	Marital status (2)	0.623	1.081	0.792	1.475
	Marital status (3)	0.243	1.188	0.890	1.587
	Years of Education	0.472			
	Years of Education (1)	0.202	1.119	0.941	1.331
	Years of Education (2)	0.760	1.029	0.855	1.238
	Years of Education (3)	0.402	1.106	0.874	1.399
	Shortage of money stops	0.000			
	Shortage of money stops (1)	0.000	0.702	0.600	0.821
	Shortage of money stops (2)	0.000	0.684	0.581	0.806
	Shortage of money stops (3)	0.000	0.651	0.533	0.796
	Current job situation	0.334			
	Current job situation (1)	0.302	1.138	0.890	1.456
	Current job situation (2)	0.313	0.773	0.468	1.275
	Number of chronic diseases	0.076			
	Number of chronic diseases (1)	0.299	1.124	0.901	1.402
	Number of chronic diseases (2)	0.039	1.249	1.011	1.541
	Number of limitations with activities of daily living (ADL) (1)	0.006	1.303	1.078	1.575
	Limitations with instrumental activities of daily living (iadl) (1)	0.028	1.197	1.019	1.406
	Limited in activities because of health	0.000			
	Limited in activities because of health (1)	0.000	0.691	0.589	0.810
	Limited in activities because of health (2)	0.000	0.535	0.443	0.646
	Hearing (1)	0.008	1.196	1.047	1.365
	Vision (1)	0.602	0.966	0.847	1.101
	Pain and level of pain	0.000			
	Pain and level of pain (1)	0.016	1.271	1.045	1.544
	Pain and level of pain (2)	0.000	1.373	1.194	1.579
	Pain and level of pain (3)	0.000	1.697	1.384	2.083
	Network satisfaction	0.039	0.955	0.914	0.998
	CASP index	0.000	0.869	0.857	0.881
	Looks after grandchildren	0.559			
	Looks after grandchildren (1)	0.753	1.031	0.854	1.244
	Looks after grandchildren (2)	0.353	1.106	0.894	1.367
	Loneliness (1)	0.000	1.962	1.666	2.311
	Number of activities in last year	0.020			
	Number of activities in last year (1)	0.015	1.319	1.055	1.649
	Number of activities in last year (2)	0.006	1.371	1.096	1.715
	Satisfaction with activities	0.000	0.933	0.901	0.965
	Ever smoked daily (1)	0.008	0.844	0.743	0.957
	At least one alcoholic beverage the last 7 days (1)	0.583	1.037	0.910	1.182
	Physical inactivity (1)	0.052	1.221	0.999	1.493
	Stayed overnight in hospital last 12 months (1)	0.005	1.248	1.068	1.460

	Received professional services (1)	0.086	1.208	0.974	1.498
	Use of internet in past 7 days (1)	0.880	1.011	0.876	1.168
	Area of building	0.040			
	Area of building (1)	0.675	1.037	0.876	1.228
	Area of building (2)	0.083	1.175	0.979	1.410
	Area of building (3)	0.011	1.258	1.054	1.501
	Type of building	0.887			
	Type of building (1)	0.895	1.013	0.832	1.234
	Type of building (2)	0.983	0.998	0.808	1.232
	Type of building (3)	0.451	0.578	0.139	2.401
**Southern**	Male or female (1)	0.000	1.936	1.710	2.191
	Marital status	0.641			
	Marital status (1)	0.246	1.186	0.889	1.584
	Marital status (2)	0.491	1.142	0.783	1.664
	Marital status (3)	0.420	1.131	0.839	1.525
	Years of Education	0.740			
	Years of Education (1)	0.349	1.059	0.939	1.193
	Years of Education (2)	0.355	1.082	0.915	1.279
	Years of Education (3)	0.730	1.046	0.809	1.354
	Shortage of money stops	0.000			
	Shortage of money stops (1)	0.000	0.641	0.550	0.747
	Shortage of money stops (2)	0.000	0.501	0.433	0.580
	Shortage of money stops (3)	0.000	0.467	0.395	0.551
	Current job situation	0.372			
	Current job situation (1)	0.160	1.286	0.905	1.827
	Current job situation (2)	0.876	1.011	0.885	1.154
	Number of chronic diseases	0.085			
	Number of chronic diseases (1)	0.872	1.016	0.838	1.232
	Number of chronic diseases (2)	0.121	1.152	0.963	1.377
	Number of limitations with activities of daily living (ADL) (1)	0.050	1.188	1.000	1.412
	Limitations with instrumental activities of daily living (iADL)(1)	0.013	1.190	1.038	1.365
	Limited in activities because of health	0.000			
	Limited in activities because of health (1)	0.000	0.750	0.648	0.868
	Limited in activities because of health (2)	0.000	0.440	0.374	0.518
	Hearing (1)	0.000	1.400	1.235	1.586
	Vision (1)	0.000	1.440	1.284	1.616
	Pain and level of pain	0.000			
	Pain and level of pain (1)	0.012	1.247	1.050	1.481
	Pain and level of pain (2)	0.000	1.437	1.274	1.622
	Pain and level of pain (3)	0.000	2.121	1.793	2.509
	Network satisfaction	0.183	0.976	0.942	1.011
	CASP index for quality of life and well-being	0.000	0.869	0.858	0.879
	Looks after grandchildren	0.005			
	Looks after grandchildren (1)	0.711	1.030	0.880	1.207
	Looks after grandchildren (2)	0.012	1.258	1.052	1.503
	Loneliness (1)	0.000	2.083	1.846	2.351
	Number of activities in last year	0.241			
	Number of activities in last year (1)	0.149	1.102	0.966	1.257
	Number of activities in last year (2)	0.944	1.005	0.866	1.168
	Satisfaction with activities	0.000	0.958	0.937	0.980
	Ever smoked daily (1)	0.031	0.881	0.784	0.989

	At least one alcoholic beverage the last 7 days (1)	0.029	0.881	0.786	0.987
	Physical inactivity (1)	0.085	1.124	0.984	1.284
	Stayed overnight in hospital last 12 months (1)	0.000	1.644	1.412	1.915
	Received professional services (1)	0.048	1.193	1.001	1.420
	Use of internet in past 7 days (1)	0.061	0.888	0.784	1.005
	Area of building	0.000			
	Area of building (1)	0.004	1.225	1.066	1.407
	Area of building (2)	0.512	0.946	0.803	1.116
	Area of building (3)	0.011	0.821	0.705	0.956
	Type of building	0.006			
	Type of building (1)	0.005	0.753	0.617	0.920
	Type of building (2)	0.165	0.851	0.679	1.068
	Type of building (3)	0.252	5.389	0.302	96.256
**Western**	Male or female (1)	0.000	1.904	1.695	2.139
	Marital status	0.137			
	Marital status (1)	0.712	1.049	0.813	1.355
	Marital status (2)	0.431	0.892	0.670	1.186
	Marital status (3)	0.545	0.920	0.702	1.206
	Years of Education	0.131			
	Years of Education (1)	0.576	0.961	0.837	1.104
	Years of Education (2)	0.181	1.111	0.952	1.296
	Years of Education (3)	0.643	0.955	0.787	1.159
	Shortage of money stops	0.000			
	Shortage of money stops (1)	0.000	0.691	0.605	0.789
	Shortage of money stops (2)	0.000	0.681	0.587	0.789
	Shortage of money stops (3)	0.002	0.713	0.576	0.884
	Current job situation	0.677			
	Current job situation (1)	0.399	0.862	0.611	1.217
	Current job situation (2)	0.811	1.026	0.830	1.268
	Number of chronic diseases	0.021			
	Number of chronic diseases (1)	0.099	1.168	0.971	1.404
	Number of chronic diseases (2)	0.007	1.273	1.069	1.516
	Number of limitations with activities of daily living (ADL) (1)	0.135	1.134	0.962	1.337
	Limitations with instrumental activities of daily living (iADL)(1)	0.001	1.273	1.102	1.471
	Limited in activities because of health	0.000			
	Limited in activities because of health (1)	0.001	0.779	0.673	0.902
	Limited in activities because of health (2)	0.000	0.608	0.514	0.719
	Hearing (1)	0.000	1.253	1.109	1.416
	Vision (1)	0.019	1.177	1.027	1.349
	Pain and level of pain	0.000			
	Pain and level of pain (1)	0.000	1.408	1.186	1.672
	Pain and level of pain (2)	0.000	1.496	1.317	1.700
	Pain and level of pain (3)	0.000	1.992	1.675	2.369
	Network satisfaction	0.170	0.970	0.929	1.013
	CASP index	0.000	0.852	0.841	0.863
	Looks after grandchildren	0.100			
	Looks after grandchildren (1)	0.287	1.089	0.931	1.273
	Looks after grandchildren (2)	0.037	1.192	1.011	1.407
	Loneliness (1)	0.000	1.990	1.721	2.301
	Number of activities in last year	0.973			

	Number of activities in last year (1)	0.818	1.033	0.786	1.356
	Number of activities in last year (2)	0.855	1.024	0.791	1.326
	Satisfaction with activities	0.000	0.926	0.893	0.960
	Ever smoked daily (1)	0.822	1.012	0.910	1.126
	At least one alcoholic beverage the last 7 days (1)	0.048	0.895	0.801	0.999
	Physical inactivity (1)	0.472	0.936	0.782	1.121
	Stayed overnight in hospital last 12 months (1)	0.015	1.174	1.032	1.336
	Received professional services (1)	0.202	1.100	0.950	1.272
	Use of internet in past 7 days (1)	0.506	1.045	0.918	1.189
	Area of building	0.609			
	Area of building (1)	0.407	0.946	0.829	1.079
	Area of building (2)	0.935	0.993	0.838	1.177
	Area of building (3)	0.229	0.913	0.788	1.059
	Type of building	0.078			
	Type of building (1)	0.014	1.414	1.072	1.866
	Type of building (2)	0.013	1.462	1.085	1.969
	Type of building (3)	0.585	1.186	0.644	2.185
**Eastern**	Male or female (1)	0.000	2.074	1.796	2.395
	Marital status	0.431			
	Marital status (1)	0.935	0.982	0.634	1.521
	Marital status (2)	0.375	0.806	0.500	1.298
	Marital status (3)	0.789	0.941	0.603	1.468
	Years of Education	0.162			
	Years of Education (1)	0.224	0.903	0.767	1.064
	Years of Education (2)	0.471	1.075	0.882	1.311
	Years of Education (3)	0.986	1.003	0.756	1.330
	Shortage of money stops	0.000			
	Shortage of money stops (1)	0.000	0.682	0.571	0.815
	Shortage of money stops (2)	0.000	0.707	0.594	0.842
	Shortage of money stops (3)	0.341	0.904	0.734	1.113
	Current job situation	0.716			
	Current job situation (1)	0.997	0.999	0.573	1.741
	Current job situation (2)	0.414	1.168	0.805	1.694
	Number of chronic diseases	0.092			
	Number of chronic diseases (1)	0.389	1.117	0.868	1.438
	Number of chronic diseases (2)	0.056	1.259	0.994	1.593
	Number of limitations with activities of daily living (ADL) (1)	0.022	1.265	1.035	1.547
	Limitations with instrumental activities of daily living (iADL)(1)	0.001	1.319	1.117	1.559
	Limited in activities because of health	0.000			
	Limited in activities because of health (1)	0.019	0.812	0.682	0.967
	Limited in activities because of health (2)	0.000	0.530	0.432	0.649
	Hearing (1)	0.000	1.347	1.157	1.567
	Vision (1)	0.000	1.399	1.217	1.608
	Pain and level of pain	0.000			
	Pain and level of pain (1)	0.000	1.456	1.183	1.792
	Pain and level of pain (2)	0.000	1.948	1.682	2.256
	Pain and level of pain (3)	0.000	2.993	2.401	3.730
	Network satisfaction	0.212	0.972	0.929	1.017
	CASP index	0.000	0.896	0.884	0.909
	Looks after grandchildren	0.102			

	Looks after grandchildren (1)	0.947	0.992	0.794	1.240
	Looks after grandchildren (2)	0.202	1.177	0.916	1.513
	Loneliness (1)	0.000	1.987	1.710	2.308
	Number of activities in last year	0.277			
	Number of activities in last year (1)	0.334	1.087	0.918	1.286
	Number of activities in last year (2)	0.109	1.155	0.968	1.377
	Satisfaction with activities	0.000	0.935	0.909	0.960
	Ever smoked daily (1)	0.000	0.774	0.676	0.886
	At least one alcoholic beverage the last 7 days (1)	0.028	1.179	1.018	1.365
	Physical inactivity (1)	0.605	0.956	0.807	1.133
	Stayed overnight in hospital last 12 months (1)	0.000	1.625	1.380	1.914
	Received professional services (1)	0.498	1.090	0.850	1.397
	Use of internet in past 7 days (1)	0.246	1.091	0.942	1.263
	Area of building	0.119			
	Area of building (1)	0.022	0.814	0.683	0.971
	Area of building (2)	0.122	0.857	0.704	1.042
	Area of building (3)	0.435	0.920	0.745	1.135
	Type of building	0.041			
	Type of building (1)	0.011	1.433	1.085	1.893
	Type of building (2)	0.007	1.534	1.122	2.095
	Type of building (3)	0.178	3.201	0.590	17.374

C.I., confidence interval; O.R., odd ratios
